# Four different frailty models predict health outcomes in older patients with stable chronic obstructive pulmonary disease

**DOI:** 10.1186/s12877-022-02750-z

**Published:** 2022-01-16

**Authors:** Dai Zhang, Wen Tang, Li-Yang Dou, Jia Luo, Ying Sun

**Affiliations:** Department of Geriatrics, Beijing Friendship Hospital, Capital Medical University, No. 95, Yong’ an Road, Xicheng District, Beijing, 100050 People’s Republic of China

**Keywords:** COPD, Frailty, Predictive accuracy, Elderly, Adverse outcomes

## Abstract

**Background:**

Frail patients with chronic obstructive pulmonary disease (COPD) face a higher risk of adverse outcomes, but there is no clear consensus on which frailty measures are most suitable for COPD patients. Herein we evaluated the ability of frailty measurements in predicting 1-year acute exacerbation, hospitalization, and mortality in older patients with COPD.

**Methods:**

A total of 302 patients [median age: 86 years (IQR: 80–90), 22.2% female] were admitted to the Department of Geriatric Medicine were prospectively enrolled in this study. Frailty status was assessed using the Fried Frailty Phenotype (FFP), Clinical Frailty Scale (CFS), Frailty Index of Accumulative Deficits (FI-CD), and Short Physical Performance Battery (SPPB). Cox proportional hazard regression and Poisson regression were used to evaluating the association of the adverse outcomes with frailty as assessed using the four instruments. The discrimination accuracy of these tools in predicting the 1-year all-cause mortality was also compared.

**Results:**

Prevalence of frailty ranged from 51% (using FFP) to 64.2% (using CFS). The four frail instruments were associated with 1-year mortality. After an average follow-up time of 2.18 years (IQR: 1.56–2.62 years), frailty as defined by four instruments (except for FI-CD), was associated with death [FFP: Hazard ratio (HR) = 3.11, 95% confidence interval (CI) 1.30–7.44; CFS: HR = 3.68, 95% CI 1.03–13.16; SPPB: HR = 3.74, 95% CI 1.39–10.06). Frailty was also associated with acute exacerbation (using FFP) and hospitalization (using FFP, CFS, and FI-CD). Frail showed a moderate predictive ability [area under the curve ranging (AUC) 0.70–0.80] and a high negative predictive value (0.98–0.99) for 1-year mortality.

**Conclusions:**

With the four different frailty assessment tools, frailty was associated with poor prognosis in older patients with stable COPD. The FFP, CFS, FI-CD, and SPPB instruments showed similar performance in predicting 1-year mortality.

**Supplementary Information:**

The online version contains supplementary material available at 10.1186/s12877-022-02750-z.

## Introduction

Frailty is a distinct biologic syndrome characterized by decreasing physiologic reserve and increasing vulnerability to minor health stressors [1–3]. Studies have shown that chronic obstructive pulmonary disease (COPD) and frailty share some common risk factors and patients’ concomitant with both COPD and frailty face a higher risk of acute exacerbations, hospitalization, and mortality [4, 5]. Physical frailty screening may be a useful tool for identifying patients that could benefit from clinical intervention. Nearly 70 frailty scales have been developed to identify frailty with varying degrees of physical, psychological, or social components [6]. According to a recent review, the Fried Frailty Phenotype (FFP) and Frailty Index (FI) are the most commonly used tools for assessing frailty in patients with stable COPD in clinics and rehabilitation centers [7]. However, there is no clear consensus as to which frailty measures are most suitable for COPD patients [8–10]. A good frailty instrument should accurately identify frailty and predict adverse outcomes. The FFP, often known as the Cardiovascular Health Study (CHS) index, is the most commonly used frailty tool. It has been demonstrated to predict mortality and adverse clinical outcomes in community-based patients with stable COPD and hospitalized and immunodeficient patients with advanced COPD, respectively [5, 11]. The Clinical Frailty Scale (CFS) is a multidimensional measure of frailty that showed a good correlation with the Frailty Index (FI) and has been validated across different clinical settings [12, 13]. The Short Physical Performance Battery (SPPB) is also a well-established tool for accessing the lower limb functional impairment in older adults, as a practical tool to assess mortality risk in patients with stable COPD [14–17]. The components of SPPB, which is a functional test evaluating standing balance, walking speed, and chair rise test, assess overlapping parameters with FFP. The recent study suggested that SPPB correlates with two widely used models of the FFP and FI [18], and can be effective to identify frailty both by the phenotype and deficit accumulation models in geriatric outpatients [19]. SPPB also has been suggested as a preferred measure in clinical trials to characterize baseline frailty by European Medicines Agency, given its prognostic value, validation status, and clinical feasibility [20].

Several studies have used frailty assessment to predict clinical outcomes in patients with COPD [4, 8, 21]. However, there is little data regarding the accuracy of various frailty instruments and this study is even rare in the COPD cohort. It remains unclear which frailty tools should be used as an outcome measure in clinical trials for patients with COPD. Therefore, we aimed to analyze the accuracy of four frailty instruments as predictors of mortality and other clinical outcomes within follow-up among older adults with stable COPD.

## Methods

### Design, setting, and participants

We prospectively studied patients aged ≥65 years with stable COPD between 1 January 2018 and 31 December 2019 at the Geriatrics Department of the Beijing Friendship Hospital, Capital Medical University. The research protocol was carried out according to the principles of the Helsinki Declaration and approved by the Research Ethics Committee of Beijing Friendship Hospital and Capital Medical University (Ethic no: 2018-P2–137-01). Written informed consent was obtained from all subjects prior to their enrolment.

Stable COPD has been defined as 1) COPD diagnosis for over a year based on GOLD 2017 guideline [22]; 2) without any change in medication for at least three months. Exclusion criteria include 1) with restrictive pulmonary diseases, acute coronary syndrome, significant heart failure, predominant neurological disability, or terminal-stage malignant tumor. 2) with the worse mental and cognitive status that could not comply with frailty assessments. Patients were classified A-D groups according to the GOLD 2017 recommendations considering symptoms [the COPD Assessment Test (CAT) and modified Medical Research Council (mMRC) dyspnea scale] and exacerbation risk.

### Measurements

At enrollment, patients underwent frailty assessment in the geriatric department using all four measures at a single time-point. Information about demographic characteristics, smoking history, body mass index (BMI), medication history, comorbidities [using the modified Charlson comorbidity index (CCI)] [23], nutrition status [using the Mini Nutritional Assessment-Short Form (MNA-SF)] [24], and functional performance [using the Katz Activities of Daily Living (ADL) scale and Lawton Instrumental Activities of Daily Living (IADL) scale] [25] were collected during face-to-face interviews or retrieved from the electronic health records at the same time.

### Frailty assessments

#### Fried frailty phenotype (FFP)

We assessed five components according to the definition of physical frailty proposed by Fried et al. [1] According to the original criteria, the components are (1) weakness; (2) slowness; (3) unintentional weight loss; (4) exhaustion, and (5) low physical activity.

Each component was classified as present (score of 1) or absent (score of 0). Patients who fulfilled ≥3 criteria were classified as frail.

#### Clinical frailty scale (CFS)

The CFS is a 9-point global assessment tool that summarizes the overall level of fitness or frailty of an older adult [12]. Each 1-score increment significantly increases the medium-term risk of mortality and institutionalization. In our study, The CFS score ≥ 5 was classified as frail, assigned by the trained physician based on preadmission functional performance, cognition, and comorbidities.

#### Frail index of accumulative deficits (FI-CD)

The FI-CD involves the accumulation of 30 or more co-morbidities, symptoms, diseases, disabilities or any deficiency in health with the idea that a greater number of health deficits indicates higher frailty [26, 27]. We constructed a 32-item FI-CD comprising medical comorbidities, presence of recent weight loss, physical and functional performance following a standardized process using information routinely captured in our comprehensive geriatric assessment (Supplement Table 1 and 2). Frailty was defined as a ratio of > 0.25 (or more than 8 of 32 deficits).

#### Short physical performance battery (SPPB)

The SPPB score was calculated as described elsewhere [14]. In brief, the SPPB contains three-time tasks: standing balance, walking speed (4 m), and five times chair stands. The standing balance has measured the participants’ ability to stand up for 10 s with their feet in three postures: side-by-side, semi-tandem, and tandem. The walking speed has measured the participants’ pace at their usual gait speed for the 4-m section. For the chair stand test, participants were instructed to stand up and sit down five times as swiftly as possible with arms folded on the chest. Each test was scored from 0 (worst performance) to 4 (best performance) based on the completion time. The total SPPB score ranged from 0 to 12, with a score ≤ 6 classified as frail [28].

### Follow-up and study outcomes

All subjects were telephonically contacted by the researchers to collect data pertaining to exacerbation frequency and hospitalization every 90 ± 5 days after enrollment. The follow-up period ended in December 2020. The primary outcome was all-cause mortality. The secondary outcomes were the frequency of AECOPD and all-cause hospitalization in the first year of follow-up.

### Statistical analysis

All continuous variables are presented as the mean ± standard deviation (SD) or median (interquartile range [IQR]/minimum value–maximum value) as appropriate, and categorical variables as frequency (percentage). Participants were classified into the following non-frail and frail groups, based on each of the four frailty instruments. The agreement between the instruments was assessed using the Kappa statistic. The association of frailty status with demographic and clinical variables was assessed using the Chi-squared of fisher’s exact test for categorical variables and one-way analysis of variance or Mann-Whitney U test for continuous variables. A time-dependent receiver operator characteristic (ROC) curve was used to assess the unadjusted predictive properties of four frailty instruments for all-cause mortality at one year. Area under the curve (AUC) of individual frailty instruments were compared to determine statistical significance. AUC > 0.70 was considered indicative of a good discriminatory value [29]. The sensitivity, specificity, positive predictive value (PPV), negative predictive value (NPV), and positive likelihood ratio (PLR) of frailty status for predicting 1-year all-cause mortality were also calculated for each frailty instrument. The association of frailty status assessed by the four instruments and poor clinical outcomes (acute exacerbation, hospitalization and all-cause death) were evaluated using Poisson regression or Cox proportional hazard regression adjusted for age, sex, medication, CCI, GOLD severity, moderate-to-severe exacerbation history, and CAT score. All statistical analyses were performed using R version 4.0.3 (The R Project for Statistical Computing, Vienna, Austria). *P*-values < 0.05 were considered indicative of statistical significance.

## Results

### Baseline characteristics and frailty prevalence

**Fig. 1:**
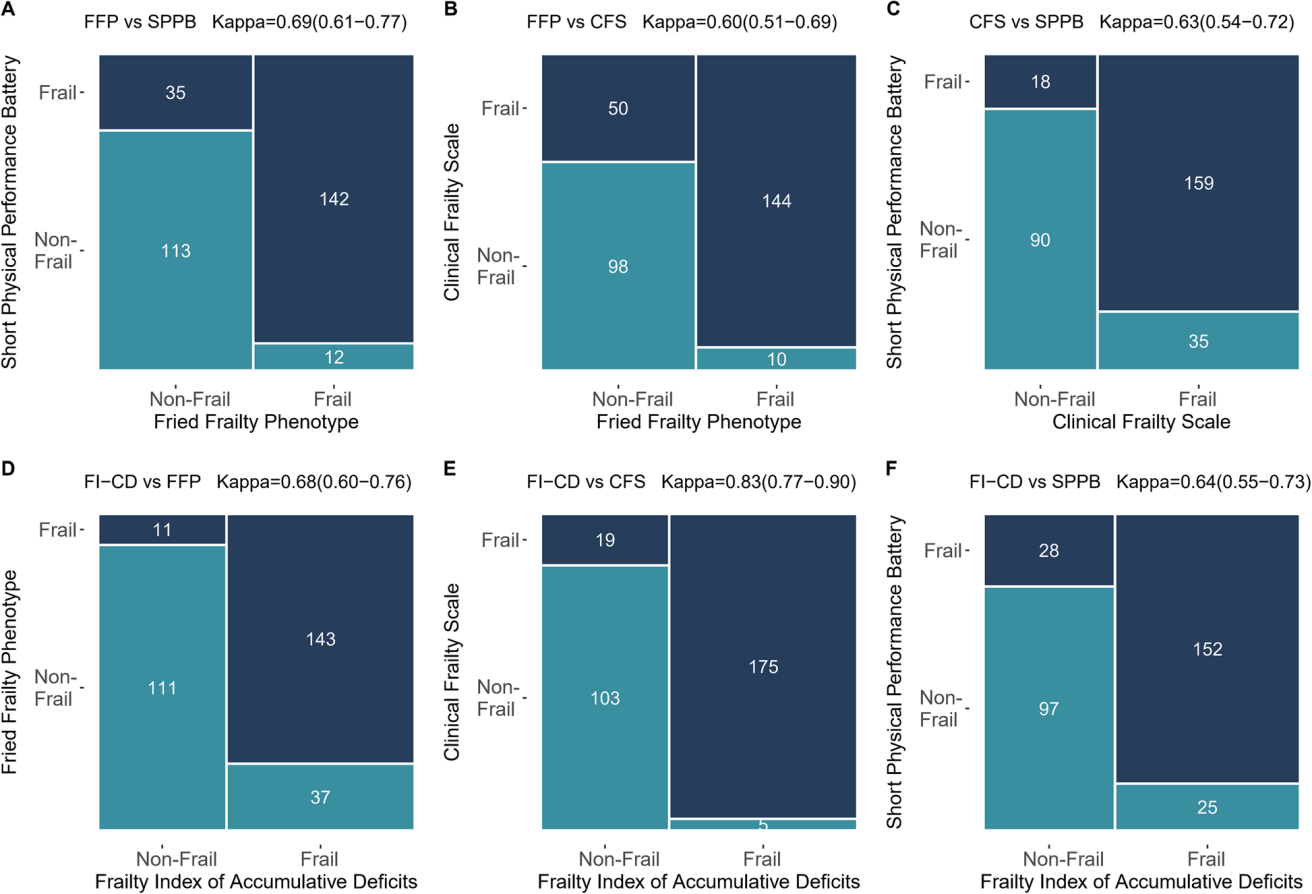
Mosaic plot representing the frequency of frailty status when evaluated by (**A**) FFP and SPPB frailty instruments, **B** FFP and CFS frailty instruments, and **C** SPPB and CFS frailty instruments, **D** FI-CD and FFP frailty instruments, **E** FI-CD and CFS frailty instruments, **F** FI-CD and SPPB frailty instruments

A total of 330 participants were included in this cohort. Of these, 16 participants (4.8%) were lost to follow-up, while 12 participants (3.6%) were excluded due to refusing to perform SPPB test. Therefore, 302 participants (22.2% female) were included in the analysis. The median age of participants was 86 [IQR: 80, 90] years (Table 1). We observed a moderate-to-substantial agreement among the four instruments (*Kappa* ranged from 0.60 to 0.68), whereas a good agreement between CFS and FI-CD (*Kappa* = 0.83) (Fig. 1). Distribution of frailty status according to any two frail measurement tools of total scores and subcomponent scores was shown in Supplement Fig. 1 and 2. Frailty prevalence estimates were 51.0% (FFP), 64.2% (CFS), 58.6% (SPPB), and 59.6% (FI-CD). Frailty, as defined by all instruments, was associated with older age, lower body mass index (BMI), more comorbidities, poorer nutritional status, poorer functional status, more symptoms (CAT or mMRC), and worse history of acute exacerbations or hospitalization when compared with non-frail status in older patients with stable COPD.

**Table 1:** Baseline demographics and clinical characteristics of non-frail and frail (assessed by the four frailty instruments) elderly COPD patients (*n* = 302)

	Total cohort	Fried Frailty Phenotype			Short Physical Performance Battery			Clinical Frailty Scale			Frailty Index of Accumulative Deficits		
		Non-frail	Frail^a^	*P*-value	Non-frail	Frail^b^	*P*-value	Non-frail	Frail^c^	*P*-value	Non-frail	Frail^d^	*P*-value
**N (%)**	302	148 (49.0)	154 (51.0)		125 (41.4)	177 (58.6)		108 (35.8)	194 (64.2)		122 (40.4)	180 (59.6)	
**Age, years, median (IQR)**	86 (80, 90)	82 (76, 88)	89 (85, 92)	< 0.001^*^	80 (75, 87)	89 (85, 92)	< 0.001^*^	78 (74, 84)	89 (85, 92)	< 0.001^*^	80 (74, 86)	89 (85, 92)	< 0.001^*^
**Sex, female n (%)**	67 (22.2)	29 (19.6)	38 (24.7)	0.356	17 (13.6)	50 (28.2)	0.004^*^	25 (23.1)	42 (21.6)	0.876	27 (22.1)	40 (22.2)	1.000
**Smoking Status, n (%)**				0.113			0.075			0.043^*^			0.021^*^
**Never**	139 (46.0)	66 (44.6)	73 (47.4)		53 (42.4)	86 (48.6)		44 (40.7)	95 (49.0)		52 (42.6)	87 (48.3)	
**Previous**	100 (33.1)	44 (29.7)	56 (36.4)		38 (30.4)	62 (35.0)		33 (30.6)	67 (34.5)		35 (28.7)	65 (36.1)	
**Current**	63 (20.9)	38 (25.7)	25 (16.2)		34 (27.2)	29 (16.4)		31 (28.7)	32 (16.5)		35 (28.7)	28 (15.6)	
**Smoking Index (pack years), median (IQR)**	5 (0, 30)	10 (0, 30)	4 (0, 30)	0.836	15 (0, 30)	3 (0, 30)	0.766	15 (0, 36)	0 (0, 30)	0.171	12 (0, 29)	3 (0, 30)	0.835
**BMI (kg/m**^**2**^**), mean (SD)**	24.2 (3.7)	25.2 (3.2)	23.2 (4.0)	< 0.001^*^	25.3 (3.3)	23.4 (3.9)	< 0.001^*^	25.1 (3.4)	23.7 (3.8)	< 0.001^*^	24.9 (3.3)	23.7 (3.9)	0.007^*^
**ADL, median (IQR)**	5 (3, 6)	6 (5, 6)	3 (2, 5)	< 0.001^*^	6 (5, 6)	4 (2, 5)	< 0.001^*^	6 (6, 6)	4 (2, 5)	< 0.001^*^	6 (6, 6)	4 (2, 5)	< 0.001^*^
**IADL, median (IQR)**	5 (2, 8)	8 (6, 8)	3 (1, 5)	< 0.001^*^	8 (6, 8)	3 (1, 5)	< 0.001^*^	8 (8, 8)	3 (1, 5)	< 0.001^*^	8 (7, 8)	3 (1, 5)	< 0.001^*^
**CCI, median (IQR)**	4 (3, 5)	3 (2, 4)	5 (3, 6)	< 0.001^*^	3 (2, 4)	4 (3, 6)	< 0.001^*^	3 (2, 4)	4 (3, 6)	< 0.001^*^	3 (2, 4)	5 (4, 6)	< 0.001^*^
**MNA-SF, median (IQR)**	12 (9, 14)	13 (12, 14)	10 (8, 12)	< 0.001^*^	13 (12, 14)	11 (8, 12)	< 0.001^*^	13 (11, 14)	11 (9, 13)	< 0.001^*^	13 (11, 14)	11 (9, 12)	< 0.001^*^
**COPD Disease Severity**													
**FEV**_**1**_**% predicted, median (IQR)**	73.5 (60.3, 85.3)	76.2 (63.9, 87.0)	70.9 (57.0, 83.5)	0.025^*^	76.2 (63.3, 87.9)	71.7 (57.5, 84.0)	0.090	76.9 (63.2, 87.1)	71.9 (58.0, 83.8)	0.076	76.9 (64.3, 88.7)	70.9 (57.7, 83.4)	0.023^*^
**GOLD severity category, n (%)**				0.114			0.299			0.167			0.100
**Mild (≥80%)**	110 (36.4)	60 (40.5)	50 (32.5)		51 (40.8)	59 (33.3)		47 (43.5)	63 (32.5)		53 (43.4)	57 (31.7)	
**Moderate (50–80%)**	157 (52.0)	76 (51.4)	81 (52.6)		62 (49.6)	95 (53.7)		50 (46.3)	107 (55.2)		56 (45.9)	101 (56.1)	
**Severe (30–49%)**	32 (10.6)	12 (8.1)	20 (13.0)		12 (9.6)	20 (11.3)		11 (10.2)	21 (10.8)		13 (10.7)	19 (10.6)	
**Very severe (< 30%)**	3 (1.0)	0 (0,0)	3 (1.9)		0 (0.0)	3 (1.7)		0 (0.0)	3 (1.5)		0 (0.0)	3 (1.7)	
**mMRC, median (IQR)**	2 (1, 3)	1 (1, 2)	2 (2, 3)	< 0.001^*^	1 (1, 1)	2 (2, 3)	< 0.001^*^	1 (0, 1)	2 (1, 3)	< 0.001^*^	1 (0, 1)	2 (2, 3)	< 0.001^*^
**mMRC ≥ 2, n (%)**	85 (28.1)	9 (6.1)	76 (49.4)	< 0.001^*^	6 (4.8)	79 (44.6)	< 0.001^*^	3 (2.8)	82 (42.3)	< 0.001^*^	5 (4.1)	80 (44.4)	< 0.001^*^
**CAT, median (IQR)**	8 (3, 14)	5 (2, 9)	12 (6, 15)	< 0.001^*^	4 (1, 9)	12 (6, 15)	< 0.001^*^	4 (1, 8)	12 (6, 15)	< 0.001^*^	4 (1, 9)	12 (6, 15)	< 0.001^*^
**CAT ≥ 10, n (%)**	131 (43.4)	35 (23.6)	96 (62.3)	< 0.001^*^	29 (23.2)	102 (57.6)	< 0.001^*^	23 (21.3)	108 (55.7)	< 0.001^*^	29 (23.8)	102 (56.7)	< 0.001^*^
**≥ 1 COPD moderate-to-severe exacerbations prior year, n (%)**	95 (31.5)	24 (16.2)	71 (46.1)	< 0.001^*^	21 (16.8)	74 (41.8)	< 0.001^*^	13 (12.0)	82 (42.3)	< 0.001^*^	17 (13.9)	78 (43.3)	< 0.001^*^
**GOLD stage**				< 0.001^*^			< 0.001^*^			< 0.001^*^			< 0.001^*^
**GOLD A, n (%)**	124 (41.1)	102 (68.9)	22 (14.3)		89 (71.2)	35 (19.8)		80 (74.1)	44 (22.7)		90 (73.8)	34 (18.9)	
**GOLD B, n (%)**	130 (43.0)	38 (25.7)	92 (59.7)		30 (24.0)	100 (56.5)		23 (21.3)	107 (55.2)		25 (20.5)	105 (58.3)	
**GOLD C, n (%)**	7 (2.3)	2 (1.4)	5 (3.2)		2 (1.6)	5 (2.8)		2 (1.9)	5 (2.6)		3 (2.5)	4 (2.2)	
**GOLD D, n (%)**	41 (13.6)	6 (4.1)	35 (22.7)		4 (3.2)	37 (20.9)		3 (2.8)	38 (19.6)		4 (3.3)	37 (20.6)	
**Medication**				0.024^*^			0.019^*^			0.119			0.198
**LAMA/LABA, n (%)**	126 (41.7)	65 (43.9)	61 (39.6)		50 (40.0)	76 (42.9)		51 (47.2)	75 (38.7)		54 (44.3)	72 (40.0)	
**2BD/BD + ICS, n (%)**	23 (7.6)	6 (4.1)	17 (11.0)		3 (2.4)	20 (11.3)		4 (3.7)	19 (9.8)		5 (4.1)	18 (10.0)	
**LAMA + LABA + ICS, n (%)**	43 (14.2)	16 (10.8)	27 (17.5)		19 (15.2)	24 (13.6)		12 (11.1)	31 (16.0)		15 (12.3)	28 (15.6)	
**None, n (%)**	110 (36.4)	61 (41.2)	49 (31.8)		53 (42.4)	57 (32.2)		41 (38.0)	69 (35.6)		48 (39.3)	62 (34.4)	

*COPD* chronic obstructive pulmonary disease, *IQR* inter-quartile range, *SD* standard deviation, *BMI* body mass index, *ADL* activity of daily living, *IADL* instrumental activity of daily living, *CCI* Charlson’s Comorbidity Index, *MNA-SF* Mini Nutritional Assessment-Short Form, *FEV*_*1*_ forced expiratory volume in 1 s, *GOLD* Global Initiative for COPD, *mMRC* modified Medical Research Council, *CAT* COPD Assessment Test, *BD* bronchodilator, *ICS* inhaled corticosteroids, *LABA* long-acting beta-agonists, *LAMA* long-acting muscarinic antagonists

^a^ Frailty was defined as Fried Phenotype scores ≥3

^b^ Frailty was defined as Short Physical Performance Battery scores ≤6

^c^ Frailty was defined as Clinical Frailty Scale scores ≥5

^d^ Frailty was defined as Frailty Index of Accumulative Deficits > 0.25

*P < 0.05

### Outcome measures

After one year of follow-up, 95 (31.5%) participants experienced AECOPD, 174 (57.6%) were hospitalized, and 28 (9.3%) died. Subjects classified as frail by any frailty instruments were at a higher risk of death in the proportional hazard models after adjusting for age, sex, medication, CCI, GOLD severity, moderate-to-severe exacerbation history, and CAT score (Table 2). Individuals classified as frail using the FFP showed a higher risk of incident acute exacerbation. Participants in the frail group based on the FFP, FI-CD, and CFS showed a greater risk of hospitalization. With an average follow-up time of 2.18 years (IQR: 1.56–2.62 years), there were 53 deaths (17.5%) in the cohort. After the full follow-up, frailty as defined by three instruments (except for FI-CD), was associated with death [FFP: Hazard ratio (HR) = 3.11, 95% confidence interval (CI) 1.30–7.44; CFS: HR = 3.68, 95% CI 1.03–13.16; SPPB: HR = 3.74, 95% CI 1.39–10.06).

**Table 2:** Comparison of adverse outcomes between frail and non-frail participants during follow-up

	Incidence Rate Ratio (95% Confidence Interval)		Incidence Rate Ratio (95% Confidence Interval)		Hazard Ratio (95% Confidence Interval)		Hazard Ratio (95% Confidence Interval)	
	1-Year Acute exacerbation		1-Year Hospitalization		1-Year Death		Death during Full Follow Up^e^	
	Adjusted Model	*P*-value	Adjusted Model	*P*-value	Adjusted Model	*P*-value	Adjusted Model	*P*-value
Fried frailty phenotype								
Non-frail	Ref		Ref		Ref		Ref	
Frail^a^	1.81 (1.13, 2.99)	0.017^*^	1.48 (1.10, 2.01)	0.011^*^	5.28 (1.39, 20.1)	0.015^*^	3.11 (1.30, 7.44)	0.011^*^
Short Physical Performance Battery								
Non-frail	Ref		Ref		Ref		Ref	
Frail^b^	1.23 (0.76, 2.05)	0.409	1.21 (0.89, 1.67)	0.229	5.99 (1.24, 28.83)	0.026^*^	3.74 (1.39, 10.06)	0.009^*^
Clinical Frailty Scale								
Non-frail	Ref		Ref		Ref		Ref	
Frail^c^	1.79 (1.00, 3.38)	0.059	1.82 (1.25, 2.69)	0.002^*^	11.32 (1.28, 100.17)	0.029^*^	3.68 (1.03, 13.16)	0.046^*^
Frailty Index of Accumulative Deficits								
Non-frail	Ref		Ref		Ref		Ref	
Frail^d^	1.69 (0.97, 3.05)	0.070	1.74 (1.22, 2.50)	0.003^*^	14.53 (1.67, 126.37)	0.015^*^	2.39 (0.82, 6.95)	0.110

Data presented are the estimated incidence rate ratios and 95% confidence intervals of the explanatory variables (1-year acute exacerbation and all-cause hospitalization) and hazard ratios and 95% confidence intervals of the explanatory variables (all-cause mortality). IRR and HR were both adjusted for age, sex, CCI, medication, GOLD severity, moderate-to-severe exacerbation history, and CAT

^a^ Frailty was defined as Fried Phenotype scores ≥3

^b^ Frailty was defined as Short Physical Performance Battery scores ≤6

^c^ Frailty was defined as Clinical Frailty Scale scores ≥5

^d^ Frailty was defined as Frailty Index of Accumulative Deficits > 0.25

^e^ the average follow-up time was 2.18 years (IQR: 1.56–2.62 years)

*P < 0.05

### Comparing individual frailty measures against the FFP

**Fig. 2:**
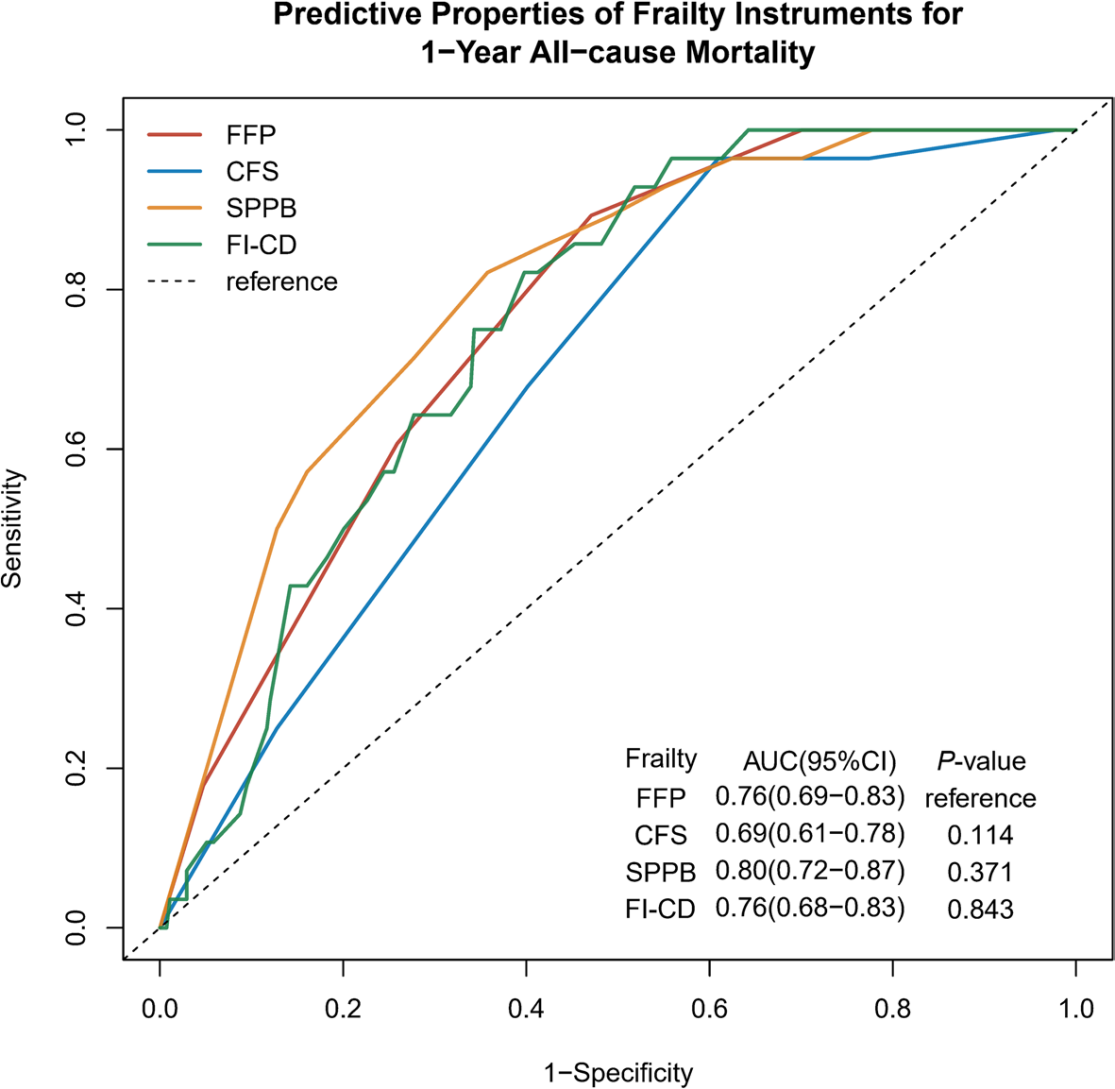
The area under the receiver operating characteristic curves for FFP, SPPB, FI-CD, and CFS predicts 1-year all-cause mortality. AUC, area under the receiver operating characteristic curves; FFP, Fried Frailty Phenotype; SPPB, Short Physical Performance Battery; FI-CD, Frailty Index of Accumulative Deficits, CFS, Clinical Frailty Scale; ROC, receiver operating characteristic curves

CFS and FI-CD showed the highest sensitivity (96%) for predicting 1-year all-cause mortality in elderly COPD patients; however, their specificities were lower (39–44%) across all measurements (Table 3). The four frailty instruments showed similar PPV and NPV for the outcomes. The ROC curves of the four frailty models were similar for all outcomes (*P* > 0.05 for all analyses) (Fig. 2). Frailty evaluated by the FFP, CFS, SPPB, and FI-CD showed a moderate performance in predicting death, ranging from 0.70 to 0.80. The addition of covariates (age, sex, medication, CCI, GOLD severity, moderate-to-severe exacerbation history, and CAT score) to the frailty instruments helped improve the ability of the models to predict death (Table 3).

**Table 3:** Results of receiver operating characteristic curve analysis showing the ability of Fried Frailty Phenotype, Short Physical Performance Battery, Clinical Frailty Scale and Frailty Index of Accumulative Deficits for predicting 1-year all-cause mortality in elderly COPD patients

	Frail	Non-frail	Sensitivity	Specificity	PPV	NPV	PLR	AUC (95% CI)	*P*-value	AUC Model^a^ (95% CI)	*P*-value
FFP	25/154	3/148	0.89	0.53	0.16	0.98	1.89	0.76 (0.69–0.83)	Ref	0.83 (0.76–0.89)	Ref
CFS	27/194	1/108	0.96	0.39	0.14	0.99	1.57	0.70 (0.61–0.78)	0.114	0.82 (0.76–0.88)	0.835
SPPB	26/177	2/125	0.93	0.45	0.15	0.98	1.69	0.80 (0.72–0.87)	0.371	0.82 (0.76–0.88)	0.779
FI-CD	27/180	1/122	0.96	0.44	0.15	0.99	1.71	0.76 (0.68–0.83)	0.843	0.82 (0.77–0.88)	0.908

*AUC* area under the curve, *PLR* positive likelihood ratio (how much more likely is a person who died during 1-year to be classified as frail than those who survived), *NPV* negative predictive value (probability that a person has no outcome when identified as non-frail), *PPV* positive predictive value (probability that a person has the outcome when identified as frail)

^a^ Adjusted model: frailty instrument plus age, sex, CCI, medication, GOLD severity, moderate-to-severe exacerbation history, and CAT

## Discussion

In the present study, we found a high prevalence of frailty, as assessed by the FFP, CFS, FI-CD, and SPPB, among older adults with stable COPD. With all four frailty assessment tools, frailty was associated with poor outcomes, such as 1-year acute exacerbation of COPD, hospitalization, or death. The FFP, CFS, FI-CD, and SPPB instruments showed comparable performance in predicting 1-year mortality.

The reported prevalence of frail among patients with COPD ranges from 6.6 to 75.5%, depending on the study population and the frailty screening tools [5, 21, 30–33]. According to a recent meta-analysis, the pooled prevalence of frail with COPD, measured by FFP, was 19% [4]. The prevalence of frail in the present study was 51.0% based on FFP, 64.2% based on CFS, 59.6% based on FI-CD, and 58.6% based on SPPB. Regardless of the instrument used, frailty in patients with stable COPD was associated with age, comorbidity, and nutrition status, the same as in the general population. Different from the study by Lahousse et al. [32], we did not observe any association of frailty (assessed with any of the four instruments) with COPD severity (as assessed by airflow limitation). It may be because Lahousse et al. compared frailty risk factors between COPD patients and subjects with normal lung function, whereas we only assessed this association in COPD patients. Consistent with Kuniaki Hirai’s research [34], the frailty assessment instruments strongly reflect the subjective symptoms (CAT/mMRC score) and frequent exacerbation of COPD rather than the pulmonary function. It suggests that frailty may be involved in the worsening of subjective symptoms and frequent exacerbations of COPD.

Currently, more than 60 instruments are available for clinical evaluation of frailty [6]; none of these instruments is considered the gold standard. The choice of a particular frailty assessment tool depends on the purpose, setting, time available, and the skills of the evaluator. The “Fried” phenotype presents a manifestation based on five predefined signs, representing evidence about clinically relevant reduced physiological function [1, 35]. FFP is based on the biological causative theory and has shown a good predictive ability for poor health outcomes across many illnesses and procedures [10]. In our study, FFP showed good performance in predicting outcomes, including AECOPD, hospitalization, and mortality. CFS is a global synthetic scale that can be readily rated using routine data from comprehensive geriatric assessment for physical frailty screening [36]. It relies upon a health professional’s evaluation of an individual’s frailty status using the descriptors as guidance, completely dependent on an individual’s ability to perform activities of daily living. The procedure is easy to implement and does not require complex questionnaires, special facilities, or any actions by patients. However, the CFS is relatively subjective, and this may affect inter-rater reliability. In the present study, the CFS identified the highest percentage of frail patients with stable COPD and showed a significant association with hospitalization and mortality. It is likely attributable to the multi-dimensional nature of the instrument, which includes comorbidity, function, and cognition [6]. In addition, older patients in our cohort presented significant functional impairment, characterized by low average ADL and IADL scores. Therefore, it is not unexpected that CFS, being guided largely by functional performance, evaluated a much higher rate of frailty. SPPB is a well-established measure for accessing reduction in physical performance in older persons, particularly muscle strength of lower extremities, the 4-m walking speed at usual pace, and balance. In our study, frailty assessed by the SPPB was associated with a 274% higher risk of mortality (HR = 3.74, 95% CI: 1.39–10.06) after the full follow-up. This conclusion is consistent with the observed association of functional capacity and muscle strength with a disability, morbidity, and mortality in older patients [17, 37].

In our study, four different frailty assessment tools showed similar moderate predictive ability for 1-year mortality. Frailty evaluation is a useful tool for predicting mortality, acute exacerbation, and hospitalization in patients with COPD. Frailty is related to a decline in multiple physical functioning and has been reported to improve by pulmonary rehabilitation [21]. Frailty screening may help identify patients who will benefit most from clinical interventions, such as rehabilitation and nutritional interventions. Currently, the consensus on the golden standard frailty instrument is absent. The suitable choice will be influenced by the scenario, the aim of the measurement, the time available, the qualification of the interviewer, and the characteristics of interviewees (e.g., community-dwelling patients with stable COPD, hospitalized patients with advanced COPD) [38]. FFP and SPPB assess physical frailty related to sarcopenia and performance, but a generalized tool summarizing multidimensional information without questionnaires and measurements, such as CFS, may better meet clinical needs. Since advanced patients with COPD commonly have features of physical frailty, associations between COPD outcomes and FFP and SPPB are predictable. While, the merit of the study is simultaneously showing impact of CFS on outcomes in COPD patients, since CFS is a global measure reflecting burden of deficit, rather than physical performance per se. Indeed, studies have shown tight correlations between validated frailty measures [18] and the current manuscript supports that either way of defining frailty (physical vs. global deficits) can be used comparably. To date, many researchers have been focusing on developing and validating models of frailties. However, we may agree that measuring and addressing frailty by any validated measures might be similarly clinically relevant as a case-finding tool, given with appropriate patient-centered care provision with the geriatric concept for frail, vulnerable population.

### Limitations

Some limitations of this study should be acknowledged, and the results should be interpreted cautiously. (1) Healthy controls were not included in this study; no additional matched or stratified analyses were performed to address confounding factors. Despite the significant relationships found between frail and COPD, no causality can be proven. (2) The study population comprised only patients with stable COPD; thus, our findings may not be generalizable to all patients with COPD, especially those with acute exacerbation or clinically advanced disease. (3) There are currently no validated frailty tools to compare with as a standard gold reference. Thus, we used the widely accepted FFP as the reference against the other frailty measurements. Given the individual-level discrepancies in frailty classification between frailty instruments as shown in Fig. 1, internal construct validity (i.e., the extent to which the assessment tool measures a construct as defined by the stated theory, i.e., “a distinct biologic syndrome characterized by decreasing physiologic reserve”), remains an important topic for future research, particularly if the goal is to advance research into its etiology. (4) We did not enroll patients with significant cognitive impairment because using FFP and SPPB requires cognitive ability to follow directions. However, evaluation of dimensions such as cognitive and social frailty is also worth considering.

## Conclusions

In this longitudinal cohort study, frailty (as evaluated by FFP, CFS, FI-CD and SPPB) was associated with 1-year AECOPD (except CFS, FI-CD, and SPPB), hospitalization (except SPPB), and mortality. The four instruments showed similar performance in predicting 1-year mortality. According to our study, the recommendations on which frailty assessment tool(s) are more or less appropriate, cannot be made. Simple and faster tools for assessing frailty improves the feasibility of their use for clinical assessment of older COPD patients.

## Supplementary Information

**Additional file 1: Supplement Table 1**. List of 32-items included in the Frailty Index of Accumulative Deficits.

**Additional file 2: Supplement Table 2**. The Frailty Index of Accumulative Deficits Continuous Variable Cut-points.

**Additional file 3: Supplement Figure 1***.* Box plot showing distributions of the Frailty Index of Accumulative Deficits according to Fried Frailty Phenotype, Short Physical Performance Battery, and Clinical Frailty Scale, respectively. In the box plot, upper, mid, and lower lines of the box denote 75th, 50th, and 25th percentiles, respectively. Upper and lower margins of whiskers denote ±1.5 interquartile range from 50th percentile. Data outside ±1.5 interquartile range from 50th percentile is shown as an outlier.

**Additional file 4: Supplement Figure 2**. Bar plot showing proportions of non-frail and frail individuals categorized using any two frailty assessment tools.

## Data Availability

Restrictions apply to the availability of data generated or analyzed during this study to preserve participant confidentiality. The corresponding author will on request detail the restrictions and any conditions under which access to some data may be provided.

## Abbreviations

ADL: Katz Activities of Daily Living
AECOPD: Acute exacerbations of COPD
AUC: Area under the curve
BD: Bronchodilator
BMI: Body mass index
CAT: COPD Assessment Test
CCI: Charlson comorbidity index
CFS: Clinical Frailty Scale
CHS: Cardiovascular Health Study
CI: Confidence intervals
COPD: Chronic obstructive pulmonary disease
FEV_1_: Forced expiratory volume in 1 s
FFP: Fried frailty phenotype
FI-CD: Frailty Index of Accumulative Deficits
FVC: Forced vital capacity
GOLD: Global Initiative for COPD
HR: Hazard ratios
IADL: Lawton Instrumental Activities of Daily Living
ICS: Inhaled corticosteroids
IQR: Interquartile range
IRR: incidence rate ratio
LABA: Long-acting beta-agonists
LAMA: Long-acting muscarinic antagonists
LR: Likelihood ratio
mMRC: modified Medical Research Council
MNA-SF: Mini Nutritional Assessment-Short Form
ROC: Receive operator characteristic
SD: Standard deviation
SPPB: Short Physical Performance Battery
