# Supplementary material for: Four different frailty models predict health outcomes in older patients with stable chronic obstructive pulmonary disease
Source: BMC Geriatr. 2022 Jan 16;22:57. doi: 10.1186/s12877-022-02750-z (PMC8761265; doi:10.1186/s12877-022-02750-z)
Supplement: Supplementary file 1 — Additional file 1: Supplement Table 1. List of 32-items included in the Frailty Index of Accumulative Deficits. [file 12877_2022_2750_MOESM1_ESM.docx]

| Variable Deficit | Deficit for Men | Deficit for Women |
| --- | --- | --- |
| Body Mass Index (BMI) | <18.5, ≥ 30 as a deficit.  25-<30 as a 'half deficit' | <18.5, ≥ 30 as a deficit.  25-<30 as a 'half deficit' |
| Grip Strength (GS in kg) | For BMI ≤ 24, GS ≤ 29  For BMI 24.1–28, GS ≤ 30  For BMI 26.1–28, GS ≤ 31  For BMI >28, GS ≤ 32 | For BMI ≤ 23, GS ≤ 17  For BMI 23.1–26, GS ≤ 17.3  For BMI 26.1–29, GS ≤ 18  For BMI>29, GS ≤ 21 |
| Physical Activity | <383kcal/week | <270kcal/week |
| Usual Pace (4.57m) | Height≤ 173cm, ≥ 7 sec  Height > 173cm, ≥ 6 sec | Height≤ 159cm, ≥ 7 sec  Height > 159cm, ≥ 6 sec |
