# Supplementary figures and images for: Four different frailty models predict health outcomes in older patients with stable chronic obstructive pulmonary disease
Source: BMC Geriatr. 2022 Jan 16;22:57. doi: 10.1186/s12877-022-02750-z (PMC8761265; doi:10.1186/s12877-022-02750-z)

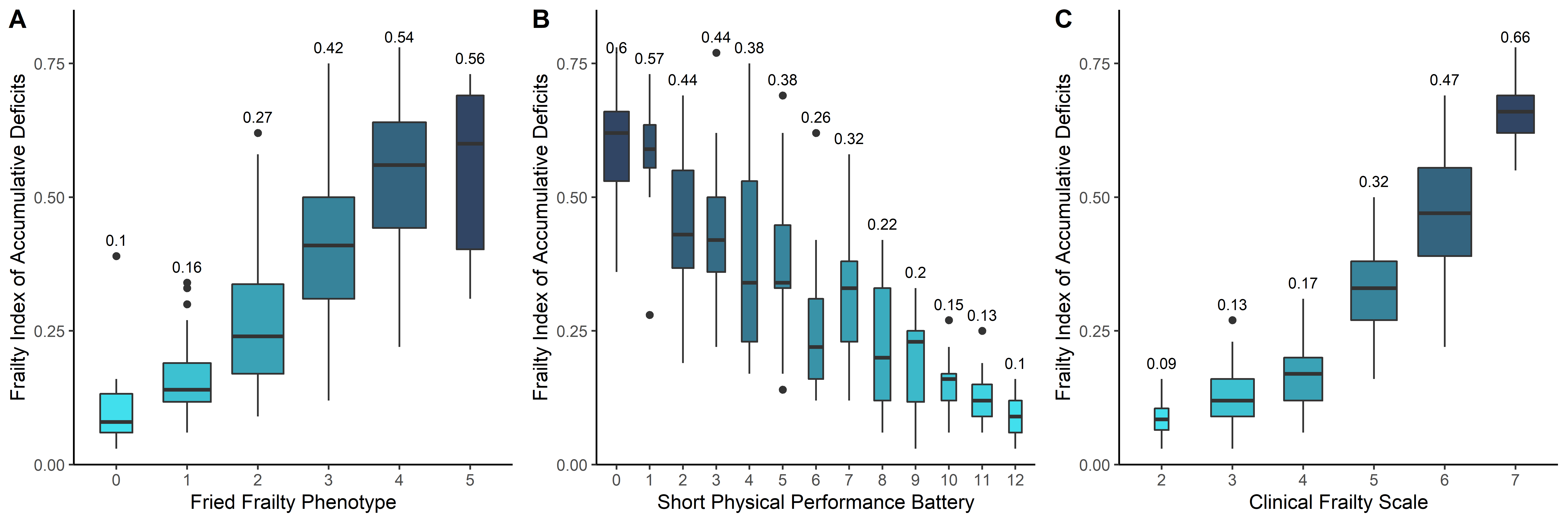

Supplement: Supplementary file 3 — Additional file 3: Supplement Figure 1. Box plot showing distributions of the Frailty Index of Accumulative Deficits according to Fried Frailty Phenotype, Short Physical Performance Battery, and Clinical Frailty Scale, respectively. In the box plot, upper, mid, and lower lines of the box denote 75th, 50th, and 25th percentiles, respectively. Upper and lower margins of whiskers denote ±1.5 interquartile range from 50th percentile. Data outside ±1.5 interquartile range from 50th percentile is shown as an outlier. [file 12877_2022_2750_MOESM3_ESM.png]
